# Perceptions of students in health and molecular life sciences regarding pharmacogenomics and personalized medicine

**DOI:** 10.1186/s40246-018-0182-2

**Published:** 2018-11-14

**Authors:** Lejla Mahmutovic, Betul Akcesme, Camil Durakovic, Faruk Berat Akcesme, Aida Maric, Muhamed Adilovic, Nour Hamad, Matthias Wjst, Oliver Feeney, Sabina Semiz

**Affiliations:** 1 Faculty of Engineering and Natural Sciences, International University of Sarajevo, Hrasnicka cesta 15, 71210 Ilidza, Sarajevo, Bosnia and Herzegovina; 2 Department of Medical Biology, Faculty of Medicine, University of Health Sciences, Istanbul, Turkey; 3 Department of Biostatistics and Medical Informatics, Faculty of Medicine, University of Health Sciences, Istanbul, Turkey; 4 Helmholtz Zentrum Muenchen, German Research Center for Environmental Health (GmbH), Ingolstaedter Landstraße 1, D-85764 Munich, Neuherberg Germany; 5 Centre of Bioethical Research and Analysis, National University of Ireland (Galway), Galway, Republic of Ireland

**Keywords:** Precision medicine, Pharmacogenomics, Education, Ethical, legal, and social issues, Survey

## Abstract

**Background:**

Increasing evidence is demonstrating that a patient’s unique genetic profile can be used to detect the disease’s onset, prevent its progression, and optimize its treatment. This led to the increased global efforts to implement personalized medicine (PM) and pharmacogenomics (PG) in clinical practice. Here we investigated the perceptions of students from different universities in Bosnia and Herzegovina (BH) towards PG/PM as well as related ethical, legal, and social implications (ELSI). This descriptive, cross-sectional study is based on the survey of 559 students from the Faculties of Medicine, Pharmacy, Health Studies, Genetics, and Bioengineering and other study programs.

**Results:**

Our results showed that 50% of students heard about personal genome testing companies and 69% consider having a genetic test done. A majority of students (57%) agreed that PM represents a promising healthcare model, and 40% of students agreed that their study program is well designed for understanding PG/PM. This latter opinion seems to be particularly influenced by the field of study (7.23, CI 1.99–26.2, *p* = 0.003). Students with this opinion are also more willing to continue their postgraduate education in the PM (OR = 4.68, CI 2.59–8.47, *p* < 0.001). Furthermore, 45% of students are aware of different ethical aspects of genetic testing, with most of them (46%) being concerned about the patient’s privacy.

**Conclusions:**

Our results indicate a positive attitude of biomedical students in Bosnia and Herzegovina towards genetic testing and personalized medicine. Importantly, our results emphasize the key importance of pharmacogenomic education for more efficient translation of precision medicine into clinical practice.

**Electronic supplementary material:**

The online version of this article (10.1186/s40246-018-0182-2) contains supplementary material, which is available to authorized users.

## Background

Personalized or precision medicine (PM) refers to an innovative approach to the disease diagnosis and treatment by considering differences in people’s genetic background, lifestyle, and environment [1–3]. Importantly, it has the potential to shape many if not all aspects of clinical care from prevention and early diagnosis to treatment of disease [4, 5]. Pharmacogenomics (PG) studies individuals’ genetic material in order to determine whether that person would benefit from a drug, require a different dose, or experience side effects, and as such is considered as an essential tool in personalized medicine [1, 6]. The successful completion of the Human Genome Project in 2003 was a first crucial step towards personalized medicine [7] and that eventually led to the Precision Medicine Initiative in the USA in 2015 to advance biomedical research in PM and facilitate its transition to clinical care [8–10]. In order to ensure the benefits of personalized diagnosis and treatment, the Food and Drug Administration (FDA) has listed about 140 drugs with pharmacogenetic/pharmacogenomic (PG) information included in their labeling [9, 11]. Importantly, the identification of genetic variants by PG tests increases the prediction regarding drug efficacy and adverse reactions [10, 12, 13]. The guidelines provided by the Pharmacogenomics Knowledgebase (PharmGKB at https://www.pharmgkb.org/) and the Clinical Pharmacogenomics Implementation Consortium (CPIC at https://cpicpgx.org/) are important educational and clinical resources for the healthcare professionals interested in introducing PG tests in their patient care.

Previous studies have shown that many physicians and pharmacists have positive attitudes towards the clinical application(s) of PG/PM [14, 15]. However, it appears that an inadequate knowledge and experience among some physicians and other healthcare professionals are the key drawbacks in more efficient clinical application of pharmacogenomics [14, 16–18], suggesting that it would be pertinent to introduce more PG topics in their professional education.

There are also many additional challenges that have to be addressed in order to facilitate its broader clinical implementation [19–21]. For example, the ethical, legal, and social implications (ELSI) of personalized medicine, such as informed consent, patient privacy, confidentiality, safety monitoring, reporting of adverse events, patient-centered practices, and other potential conflicting interests, have been addressed in existing bioethical analyses [20, 22–24]. However, further ethical implications associated with personalized medicine are emerging, for example, where it can be observed that different ethnicities may have different response to drugs [25–27]. Furthermore, some bioethicists are also concerned about the increased trend of human genome sequencing, processing, and storing of databases, private genetic bio/databanks, as well as about the increasingly popular direct-to-consumer (DTC) genetic tests, incidental (unsolicited) non-PG findings, potential health disparities, and other socioeconomic barriers in a broad PG application [28–32].

It is also important to mention here an issue which is often overlooked and that is of the fairness (or lack thereof) related to how the benefits of pharmacogenetic research, such as an increasing number of genomic advances relevant to disease prevention, diagnosis, and treatment [3] together with the decreasing costs of genetic testing [33], are currently being shared at the global level. A recent study performed by Manolio et al. [34] identified the major barriers to global implementation of genomic medicine, including high costs and/or lack of reimbursement and limited access to reliable standardized genotyping or sequencing platforms. Previous studies concluded that countries with limited research sources should have a chance to also express their opinions in making global decisions regarding public access and benefits from the commercialized products, such as (pharmaco) genetic tests [35]. Transnational collaboration through the large research consortia and sharing information in the areas of health information technology, pharmacogenomics, education, professional development, and policy and regulatory issues seems to be pertinent for the future efficient clinical implementation of personalized medicine at the global level [34]. Increasing evidence is demonstrating different views and attitudes regarding PG testing, including patients’ concerns about privacy, discrimination, quality of care, and value of the relationship between patient and physician [32]. Recently, several survey-based studies have been performed in order to assess the knowledge and awareness of health science students in the area of pharmacogenomics, personalized medicine, and bioethics [36–39]. Results of these studies demonstrated that health science students’ knowledge and appreciation of PG is very important for optimal patient care. They should have the necessary skills and knowledge to make more rational therapy decisions based on patients’ genetic information [38].Thus, education and raising awareness of biomedical students are of key importance for the future practice of precision medicine.

More than a decade ago, the International Society of Pharmacogenomics proposed recommendations regarding PG education standards to the medical, pharmacy, and health schools globally [40]. Consequently, many medical and pharmacy schools around Europe adopted these recommendations and included PG topics in their curricula [41–45], while only a few programs have been evaluated.

This is the first study of students’ perception of pharmacogenomics in Bosnia and Herzegovina (BH). Although there are a few pharmacogenetic studies that have been performed in BH [46–50], an inadequate understanding of pharmacogenomics, expertise, and limited resources of the healthcare system in this middle-income country appears to represent the major challenges in clinical application of PG. Since the views of BH students have not been investigated yet on this topic, it is pertinent to understand the current status and needs for pharmacogenomic education in order to develop appropriate educational and training programs among professionals and students in health and molecular life sciences. Here we investigate the awareness and attitudes of health science (medical, pharmacy, health studies) and molecular life science (genetics and bioengineering) students in BH towards genetic testing, pharmacogenomics, and personalized medicine. As a second outcome, different ethical, legal, and social issues (ELSI) of personalized treatment have been also investigated. While health and molecular life science university students are not representative of the population as a whole, given their roles as the future medical doctors, nurses, pharmacists, and other health care professionals in BH society, it is important to capture their views.

## Methods

This descriptive, cross-sectional study was done using online and hard copy questionnaires (survey is accessible as Additional file 1) between the second and eighth week of the spring semester in February and March 2016. Surveys were distributed by the teachers during a class or they were accessed online. Eligible participants included current students from several universities in Bosnia and Herzegovina (BH) located in four different cities, including Sarajevo, Tuzla, Mostar, and Bihac. The total number of 559 students participating in the survey involved students from the Faculty of Pharmacy, Faculty of Medicine, Faculty of Health Studies (FHS), Genetics and Bioengineering (GBE), as well as students from other non-health science (HS) and non-molecular life science (MLS)-related faculties.

The survey consisted of four clusters from a total of 33 questions on the following: (i) demographic and professional characteristics of the participants, (ii) participants’ diseases and treatment, (iii) awareness and attitudes towards genetic testing and personalized medicine approach, and (iv) challenges towards genetic tests, PG, and its clinical application. Key definitions of genetic testing, personalized medicine, and pharmacogenomic/pharmacogenetic test were provided to the participants in the instruction section of the survey. All survey questions were consistent across all participating faculties. The survey included yes/no/I do not know (not sure) questions. In addition, the survey asked for levels of agreement with various statements using a Likert scale (i.e., agree, disagree, no opinion, neutral) and also offered multiple choice questions. Before being sent to students for the study, this questionnaire was reviewed by three experts from various backgrounds (clinical genetics, genomics, genetic counseling, genetic education, ethics, and social sciences). Along with the survey, an introductory cover page was attached describing the purpose and objectives of the study and inviting the students to participate in the study. Participants were assured their identity and all data are confidential. Participation was voluntary, and the study was approved by the Ethical Committee of the International University of Sarajevo.

### Statistical analysis

All categorical variables including participants’ demographics, professional information, and answers to questions regarding the perceptions about PG and PM were expressed as frequencies and percentages. Descriptive analysis was performed by using chi-square test and ANOVA for categorical variables. In addition, the binary logistic regression was performed in order to assess association between question of interest and hypothetically related covariates, while adjusting for age, gender, and level of education. In model I, we present this association prior to adjustment; in model II, we analyzed this association adjusted for age and gender, while model III additionally included an adjustment for the level of education. Odds ratio (OR) and corresponding 95% confidence intervals (CI) were computed, using a significance level of 5% for all statistical tests. Statistical analysis was performed by using IBM Statistical Package for Social Sciences (IBM SPSS®23).

## Results

### Participants’ characteristics

Table 1 summarizes the students’ demographics characteristics and professional information. The response rate was calculated for all students who completed the survey (*N* = 559, 10% response rate), including students from the Faculty of Pharmacy (*N* = 183), students from the Faculty of Medicine (*N* = 158), students from the Faculty of Health Studies (*N* = 64), students from the Genetics and Bioengineering (*N* = 66), and 88 students from other non-HS- and non-MLS-related study programs (architecture; psychology; industrial, mechanical, and electrical engineering; computer sciences; law; political sciences; and visual arts) (*N* = 88). The majority of participants were female (71%) and undergraduate students (*N* = 398, 84%), while 12% (*N* = 60) were attending Master and 3% (*N* = 13) PhD programs, with age ranging from 19 to 26 years old (86%).

**Table 1:** Students’ demographic characteristics and professional information

	Total	Faculty of Pharmacy	Faculty of Medicine	Genetics and Bioengineering	Faculty of Health Studies	Non-ML&HS faculties	p*
Gender							
Male	153 (29)^a^	25 (15)	46 (29)	16 (26)	19 (29)	47 (54)	< 0.01
Female	382 (71)	140 (85)	110 (71)	46 (74)	46 (71)	40 (46)	
Total	535	165	156	62	65	87	
Age							
< 19	37 (6.8)	–	–	19 (30)	10 (15)	8 (9)	< 0.01
19–26	465 (86)	141 (85)	154 (97)	44 (70)	54 (82)	72 (82)	
26–40	37 (6.8)	24 (14)	4 (2)	–	2 (3)	7 (8)	
41–50	1 (0.2)	–	–	–	–	1 (1)	
51–60	1 (0.2)	1 (1)	–	–	–	–	
> 60	1 (0.2)	–	1 (1)	–	–	–	
Total	542	166	159	63	66	88	
Level of education							
Less than high school	3 (1)	–	1 (1)	–	–	2 (2)	< 0.01
BSc	398 (84)	89 (63)	129 (95)	56 (97)	60 (97)	64 (82)	
MSc	60 (12)	46 (33)	4 (3)	2 (3)	2 (3)	6 (8)	
PhD	13 (3)	6 (4)	1 (1)	–	–	6 (8)	
Total	474	141	135	58	62	78	

*ML&HS*, Molecular Life and Health Science;

*Chi-square test, Bonferroni-adjusted *p* values

^a^Percentage (%)

### Students’ attitudes towards pharmacogenetic testing and personalized medicine

Participants’ responses to almost all survey questions regarding their awareness and attitudes towards genetic testing, pharmacogenomics, personalized medicine, and corresponding ELSI are shown in the tables, with selected ones that are further elaborated in the discussion. As shown in Table 2, about 30–40% of participants from medicine, pharmacy, health studies, and genetics and bioengineering experienced that a particular drug did not work for them, while about 15–25% of these students had an adverse drug reaction. When asked about personal genome testing companies, about half of the participants from all HS and MLS faculties responded that they have heard about these companies and the majority of students (69%) showed an interest in having a genetic test done. About 40% of students would also consider contacting a personal genome testing company and ordering a PG test. The majority of students (70%) believe that genes moderately influence their health, with 13% of them thinking that genes completely affect it. When asked would they take the drug if a PG test revealed that prescribed drug would either be ineffective or cause severe side effects, about 40% of all students responded that they would accept the test result and take the drug only if the disease might be life-threatening (Table 2). Furthermore, more than half of all students (57%) agreed that personalized medicine represents a promising healthcare model.

**Table 2:** Students’ attitudes towards pharmacogenetic testing and personalized medicine

	Total	Faculty of Pharmacy	Faculty of Medicine	Genetics and Bioeng.	Faculty of Health Studies	Non- ML&HS faculties	p*
Have you heard about personal genome testing companies?							
Yes	271 (50)^a^	95 (57)	101 (64)	29 (44)	23 (37)	23 (27)	< 0.01
No	136 (26)	39 (23)	26 (16)	25 (38)	22 (36)	24 (18)	
Do not know	131 (24)	32 (20)	31 (20)	12 (18)	17 (27)	39 (31)	
Total	538	166	158	66	62	86	
To what extent do you think genes influence your health?							
Completely	74 (13)	30 (18)	18 (11)	8 (13)	7 (11)	11 (13)	< 0.01
Moderately	375 (69)	129 (78)	132 (80)	43 (68)	36 (56)	35 (41)	
Not at all	37 (7)	1 (1)	10 (6)	4 (6)	1 (2)	21 (24)	
Do not know	58 (11)	6 (3)	5 (3)	8 (13%)	20 (31)	19 (22)	
Total	544	166	165	63	64	86	
Would you consider having a genetic test done to find out what illnesses you might develop in the future?							
Yes	374 (69)	138 (83)	101 (64)	45 (68)	38 (59)	52 (60)	< 0.01
No	110 (21)	20 (12)	36 (23)	15 (23)	20 (31)	19 (22)	
Not sure	56 (10)	8 (5)	20 (13)	6 (9)	6 (10)	16 (18)	
Total	540	166	157	66	64	87	
Do you agree that personalized medicine represents a new and promising healthcare model?							
Agree	295 (57)	116 (70)	83 (52)	31 (74)	24 (37)	41 (48)	< 0.01
Disagree	62 (12)	14 (8)	17 (11)	1 (2)	10 (16)	20 (23)	
Not sure	160 (31)	37 (22)	58 (37)	10 (24)	30 (47)	25 (29)	
Total	517	167	158	42	64	86	
Have you ever had an adverse drug reaction?							
Yes	91 (17)	41 (25)	22 (14)	8 (13)	10 (16)	10 (11)	< 0.01
No	328 (61)	82 (51)	112 (71)	45 (69)	42 (67)	47 (54)	
Do not know	89 (16)	38 (23)	20 (13)	10 (15)	8 (13)	13 (15)	
I have never taken any medication	29 (6)	2 (1)	4 (2)	2 (3)	3 (5)	18 (20)	
Total	537	163	158	65	63	88	
Have you ever found that a particular drug did not work for you?							
Yes	184 (34)	67 (41)	47 (30)	26 (39)	25 (39)	19 (22)	
No	226 (42)	69 (42)	76 (49)	25 (38)	25 (39)	31 (35)	
Do not know	90 (17)	27 (16)	26 (18)	11 (17)	7 (11)	19 (22)	
I have never taken any medication	37 (6)	2 (1)	5 (3)	4 (6)	7 (11)	19 (22)	
Total	537	165	154	66	64	88	
If a PG test revealed that prescribed drug would either be ineffective or cause severe side effects, would you take the drug anyway?							
Take the drug anyway	62 (12)	17 (10)	38 (23)	6 (10)	9 (14)	2 (2)	< 0.01
Accept the test result and not take the drug	117 (22)	34 (20)	27 (16)	20 (30)	20 (32)	16 (19)	
Accept the test result and take the drug only if the disease might be life-threatening	207 (39)	79 (48)	61(37)	24 (36)	15 (24)	28 (33)	
Not sure	150 (28)	36 (22)	39 (24)	16 (24)	19 (30)	40 (46)	
Total	536	166	165	66	63	66	
Would you consider contacting a personal genome testing company and ordering a PG test for yourself?							
Yes	211 (39)	85 (52)	54 (33)	30 (45)	28 (44)	20 (23)	0.01
No	117 (22)	24 (14)	39 (24)	14 (21)	17 (27)	21 (24)	
Do not know	210	57 (34)	69(43)	22 (34)	18 (29)	45 (53)	
Total	538	166	162	66	63	86	

*ML&HS* Molecular Life and Health Science

*Chi-square test, Bonferroni-adjusted *p* values

^a^Percentage (%)

The level of awareness about companies offering PG tests appears to be similar between medicine and pharmacy students (not significantly different; see Additional file 2). Students from the Faculty of Health Studies are less aware of genome testing companies than their colleagues from the medicine and pharmacy (*p* = 0.010 and *p* = 0.025, respectively). A significantly lower number of these students agreed that PM represents the new and promising healthcare model as compared to the pharmacy and genetics students (*p* < 0.01 and *p* = 0.01, respectively; Additional file 3).

As shown in Table 2, respondents from the other non-health and non-molecular life sciences-related studies are generally aware that genes affect their health, and about half of them agree that personalized medicine represents a new and promising healthcare model. Furthermore, the majority of these students (60%) from non-HS and non-MLS faculties would consider having genetic test done to find out what illnesses they might develop in the future. However, their awareness of personal genome testing companies is significantly lower as compared to their peers from medicine, pharmacy, and genetics (*p* < 0.01, *p* < 0.01, and *p* = 0.02, respectively; Additional file 2). In addition, as shown in Table 2, a lower number of these students (23%) from the non-health and non-molecular life science studies would consider contacting a personal genome testing company to order a PG test, as compared to the number of students from pharmacy (52%), health sciences (44%), and GBE (45%).

Our results of logistic regression analysis, performed to determine which independent variables were the strongest predictors of the specific students’ responses, demonstrated belief that genes influence health in moderate to complete extent. Students with this belief would consider having a genetic test done to find out which illnesses they might develop in the future (OR = 3.02, CI 1.16–7.85, *p* = 0.024) (Table 3). This association does not appear to be affected by age, gender, and/or levels of education. Furthermore, our results demonstrated that those students who agreed that personalized medicine represents a new and promising healthcare model were also willing to do a genetic test, as compared to those who think the opposite (OR = 3.11, CI 1.60–6.06, *p* = 0.001). In addition, students who are ready to make necessary changes in their lifestyle to reduce disease risk would also consider having a genetic test done to know their genetic tendency to develop a disease (OR = 0.198, CI 0.114–0.283, *p* = 0.001). Our results also indicated that students’ response on how much money they would be willing to spend to examine the effectiveness of a specific drug by using PG test was associated with their family monthly income (OR = 0.229, CI 0.065–0.392, *p* = 0.006), regardless of the field of their study (OR = 0.033, CI − 0.075–0.141, *p* = 0.543). This association does not seem to be affected by age, gender, and/or levels of education.

**Table 3:** Students’ attitudes towards pharmacogenomics and personalized medicine

Would you consider having a genetic test done to find out what illnesses you might develop in the future?			
	Model I	Model II	Model III
	OR (CI)	OR (CI)	OR (CI)
Q1			
To what extent do you think that genes influence your health?	3.02 (1.16–7.85) *p* = 0.024	3.04 (1.17–7.92) *p* = 0.023	2.81 (1.07–7.36) *p* = 0.035
Q2			
Do you agree that PM represents a new and promising healthcare model?	3.11 (1.60–6.06) *p* = 0.001	3.11 (1.60–6.06) *p* = 0.001	3.14 (1.61–6.15) *p* = 0.001
Q3			
If you know your genetic tendency to develop a disease, would you be ready to make necessary changes in your lifestyle, to reduce disease risk?	0.198 (0.114–0.283) *p* = 0.001	0.195 (0.109–0.281) *p* = 0.001	0.191 (0.102–0.210) *p* = 0.001

Model I: without adjustment; model II: adjusted to age and gender; model III: adjusted to age, gender, and level of education

Answer as a reference for Q1: completely; answer as a reference for Q2 and Q3: yes

### Importance of pharmacogenomics education

Results presented in Table 4 demonstrated similar opinion between medical, pharmacy, and health studies students regarding their study curriculum and future plans related to PG. When asked about their study curriculum, 44% of pharmacy students, 51% of medical students, and 61% of health studies students agreed that their study program is well designed for understanding PG. However, we found that only 20% of GBE students share this opinion, while 71% of them believe that PG should be an important part of their study curriculum (*p* < 0.01, see Additional file 4). About 30% of respondents are mostly interested to learn about pharmacogenomics in general, its clinical examples and benefits, while about 20% of students would like to learn more about its corresponding ethical, legal, and social issues. More than half of GBE students (55%) would like to continue their postgraduate education in the field of personalized medicine. Similarly, 74% of health studies students, 65% of pharmacy students, and 48% of medical students are also interested to continue their education in personalized medicine, which was significantly different as compared to the students from non-ML and non-HS study programs (*p* < 0.01; see Additional file 5). A similar finding was observed related to the students’ opinion of PG position in their study curriculum, where significantly more medical, pharmacy, and genetics students agreed about an importance of PG as compared to their peers from other study programs (*p* < 0.01).

**Table 4:** Students’ opinion regarding the study curriculum and their future plans in pharmacogenomics

	Total	Faculty of Pharmacy	Faculty of Medicine	Genetics and Bioengineering	Faculty of Health Studies	Non- ML&HS faculties	p*
Do you think that the curriculum of your study program is well designed for understanding pharmacogenomics?							
Agree	219 (40)^a^	74 (44)	78 (51)	15 (20)	37 (61)	15 (18)	< 0.01
Disagree	185 (34)	71 (43)	40 (26)	25 (32)	17 (28)	32 (37)	
Not sure	139 (26)	21 (13)	36 (23)	37 (48)	7 (11)	38 (45)	
Total	543	166	154	77	61	85	
Pharmacogenomics should be an important part of my study curriculum.							
Agree	204 (38)	65 (39)	61 (39)	46 (71)	7 (11)	25 (29)	< 0.01
Disagree	112 (21)	86 (52)	8 (5)	1 (1)	–	17 (20)	
Not sure	318 (41)	15 (9)	86 (56)	18 (28)	55 (89)	44 (51)	
Total	534	166	155	65	62	86	
Would you like to continue your postgraduate education in the field of personalized medicine?							
Yes	275 (53)	107 (65)	74 (48)	36 (55)	45 (74)	13 (16)	< 0.01
No	97 (18)	17 (10)	41 (27)	6 (9)	10 (16)	32 (39)	
Do not know	146 (29)	40 (25)	39 (25)	24 (36)	6 (10)	37 (45)	
Total	527	164	154	66	61	82	

*ML&HS*, Molecular Life and Health Science

*Chi-square test, Bonferroni-adjusted *p* values

^a^Percentage (%)

Our findings presented in Table 5 showed that students who believe that their study curriculum is well designed agreed that PG should be an important part of their study curriculum (OR = 0.54, CI 0.33–0.87, *p* = 0.01). They also believe that in their future practice they should be able to identify patients that could benefit from genetic testing (OR = 0.48, CI 0.31–0.75, *p* = 0.001) as well as to be able to answer patients’ questions regarding PG and PM (OR = 1.70, CI 1.01–2.82, *p* = 0.047).

**Table 5:** Students’ attitudes towards continued education in pharmacogenomics

Do you think that the curriculum of your study program is well designed for understanding PG?			
	Model I	Model II	Model III
	OR (CI)	OR (CI)	OR (CI)
Q1			
PG should be an important part of my study curriculum.	0.54 (0.33–0.87) *p* = 0.012	0.54 (0.33–0.88) *p* = 0.014	0.75 (0.43–1.30) *p* = 0.307
Q2			
In my future practice, I should be able to identify patients that could benefit from genetic testing.	0.48 (0.31–0.75) *p* = 0.001	0.48 (0.31–0.70) *p* = 0.002	0.57 (0.35–0.91) *p* = 0.020
Q3			
In my future practice, I should be able to answer patients’ questions regarding PG and personalized medicine.	1.70 (1.01–2.82) *p* = 0.047	1.71 (1.02–2.87) *p* = 0.044	1.51 (0.87–2.62) *p* = 0.146
Q4			
In my future practice, I should be able to identify drugs that would require PG testing prior to their administration to the patient.	0.043 (0.011–0.098) *p* = 0.121	0.050 (0.006–0.107) *p* = 0.079	0.049 (0.007–0.106) *p* = 0.086
Q5			
What is your field of study?	3.94 (1.37–11.33) *p* = 0.011	7.23 (1.99–26.2) *p* = 0.003	7.23 (1.99–26.2) *p* = 0.003

Model I: without adjustment; model II: adjusted to age and gender; model III: adjusted to age, gender, and level of education

Answer as a reference for Q1: agree; answer as a reference for Q2: agree; answer as a reference for Q3: agree; answer as a reference for Q4: agree; answer as a reference for Q5: Genetics and Bioengineering

As shown in Table 6, our results suggest that the field of study significantly affects students’ attitudes related to their study curriculum (OR = 3.94, CI 1.37–11.33, *p* = 0.011) as well as influences students’ wish to continue their postgraduate education in the area of personalized medicine. As compared to other respondents, it appears that the highest number of GBE students would like to continue their postgraduate education in this field (OR = 14.7 CI 4.31–49.9, *p* < 0.001, upon adjustment to students’ gender, age, and level of education). Furthermore, our results suggest that students who believe that their study program is well designed to provide them with an adequate understanding of PG are also more willing to continue their postgraduate education in the area of personalized medicine (OR = 4.68, CI 2.59–8.47,*p* < 0.001). Similarly, it appears that these students also believe that PG should be an important part of their study curriculum (OR = 1.79 CI 1.01–3.19, *p* = 0.045), and this opinion is particularly affected by the level of education (OR = 2.40 CI 1.28–4.48, *p* = 0.006).

**Table 6:** Students’ attitudes towards continued education in pharmacogenomics

Would you like to continue your postgraduate education (master, PhD, specialization) in the field of personalized medicine?			
	Model I	Model II	Model III
	OR (CI)	OR (CI)	OR (CI)
Q1			
PG should be an important part of my study curriculum.	1.73 (0.99–3.02) *p* = 0.056	1.79 (1.01–3.19) *p* = 0.045	2.40 (1.28–4.48) *p* = 0.006
Q2			
Do you think that the curriculum of your study program is well designed for understanding PG?	4.68 (2.59–8.47) *p* < 0.001	4.71 (2.59–8.57) *p* < 0.001	4.27 (2.28–7.99) *p* < 0.001
Q3			
What is your field of study?	14.7 (4.31–49.9) *p* < 0.001	14.1 (3.94–50.6) *p* < 0.001	16.05 (4.05–63.6) *p* < 0.001

Model I: without adjustment; model II: adjusted to age and gender; model III: adjusted to age, gender, and level of education

Answer as a reference for Q1: agree; answer as a reference for Q2: agree; answer as a reference for Q3: Genetics and Bioengineering

Furthermore, as shown in Table 7, our results demonstrated an interesting difference in attitude between pharmacy students from Sarajevo and Tuzla. A significantly higher number of pharmacy students at the University of Sarajevo, who have an elective course “Pharmacogenomics and Personalized Therapy” included in their study curriculum, believe that genes influence their health (*p* = 0.011), consider having genetic test done (*p* < 0.05), and agree that PM represents a new and promising healthcare model (*p* < 0.001), as compared to their colleagues from the Faculty of Pharmacy in Tuzla whose curriculum seems to cover PG education only as a few topics built into other coursework. In addition, the higher number of pharmacy students from Sarajevo agree that PG should be an important part of their study curriculum (*p* < 0.001). All surveyed pharmacy students from the University of Tuzla disagree that PG should be an important part of their study curriculum as opposed to only 6% of pharmacy students from the University of Sarajevo who believe that PG is not essential for their education.

**Table 7:** Pharmacy students’ awareness and opinion regarding genetic tests and pharmacogenomics

	Total	Faculty of Pharmacy, University of Sarajevo	Faculty of Pharmacy, University of Tuzla	p*
Have you heard about personal genome testing companies?				
Yes	95 (57)^a^	53 (62)	42 (52)	0.261
No	39 (24)	17 (20)	22 (27)	
Do not know	32 (19)	15 (18)	17 (21)	
Total	166	85	81	
To what extent do you think genes influence your health?				
Completely	30 (18)	20 (24)	10 (12)	0.011
Moderately	129 (78)	64 (75)	65 (80)	
Not at all	1 (1)	–	1 (1)	
Do not know	6 (4)	1 (1)	5 (6)	
Total	166	85	81	
Would you consider having a genetic test?				
Yes	85 (51)	51 (60)	34 (42)	0.047
No	24 (14)	9 (11)	15 (18)	
Not sure	57 (34)	25 (29)	32 (40)	
Total				
Do you agree that personalized medicine represents a new and promising healthcare model?				
Agree	116 (70)	78 (92)	38 (46)	*p* < 0.001
Disagree	14 (8)	2 (2)	12 (15)	
Not sure	37 (22)	5 (5.9)	32 (39)	
Total	167	85	82	
Pharmacogenomics should be an important part of my study curriculum.				
Agree	65 (39)	65 (76)		*p* < 0.001
Disagree	86 (52)	5 (5.9)	81 (100)	
Not sure	15 (9)	15 (18)		
Total	166	85	81	
Would you like to continue your postgraduate education in the field of personalized medicine?				
Yes	107 (64)	31 (36)	76 (94)	*p* < 0.001
No	19 (11)	16 (19)	3 (4)	
Do not know	40 (24)	38 (45)	2 (2)	
Total	166	85	81	

*Chi-square test, Bonferroni-adjusted *p* values

^a^Percentage (%)

### Students’ awareness about the ethical, legal, and social implications (ELSI)

Our results showed that about 45% of all students participating in our survey are aware of different ethical aspects of genetic testing, ranging from 27% of students at the Faculty of Health Studies to 54% of pharmacy students (*p* < 0.01, Table 8 and Additional file 6). The highest percentage (46%) of all respondents believed that patient privacy is the most related ethical issue to pharmacogenetic testing, while 18% believed that the key issue is data confidentiality (*p* < 0.01). Other ethical issues, such as incidental findings, racial issues, and stigma, were selected by 9%, 5%, and 4% of students, respectively. Our results revealed that 44% of students are worried about the possibility that PG test results may be passed to the unauthorized persons, and this opinion was shared similarly across different faculties (no significant difference). When asked which of the healthcare professionals should have an access to their PG information, 75% of students believe that a physician, 50% of students selected a genetic counselor, while 35% of them believe that a pharmacist should have this information. Furthermore, approximately one third of the respondents believe that they would be disadvantaged at work or job seeking in a case of unfavorable results of genetic test. Our analysis of questions related to the social issues showed that about half of participating students would not feel “helpless” or “pessimistic” (49%) nor they would feel “different” or “inadequate” (50%) in case of the unfavorable test results. Students’ answers regarding all above ELSI were similar across all participating disciplines (no significant difference). Our results presented in Table 9 showed that students who are worried about the possibility that PG test may reveal that they have additional risk factors for other diseases would also feel “different” and “inadequate” (OR = 2.48, CI 1.34–4.60, *p* = 0.004). A similar finding was demonstrated upon adjustment to students’ age, gender, and level of education (OR = 2.15, CI 1.13–4.10, *p* = 0.020).

**Table 8:** Students’ awareness and opinion regarding the ethical, legal, and social implications (ELSI)

	Total	Faculty of Pharmacy	Faculty of Medicine	Genetics and Bioengineering	Faculty of Health Studies	Non- ML&HS	p*
Ethical							
Are you aware of the different ethical aspects of genetic testing?							
Yes	244 (45)^a^	90 (54)	50 (36)	28 (43)	17 (27)	26 (30)	< 0.01
No	155 (29)	32 (19)	60 (43)	22 (34)	32 (50)	33 (38)	
Not sure	139 (26)	44 (27)	30 (21)	15 (23)	15 (23)	27 (32)	
Total	538	166	140	65	64	86	
What ethical issues do you believe might be related to genetic or PG testing?							
Patient privacy	237 (46)	88 (40)	63 (34)	29 (38)	30 (41)	27 (29)	< 0.01
Data confidentiality	91 (18)	82 (37)	56 (30)	14 (18)	13 (18)	8 (8)	
Racial issues	27 (5)	10 (5)	4 (3)	2 (3)	12 (17)	9 (10)	
Stigma	20 (4)	7(3)	17 (9)	–	–	3 (3)	
Incidental findings	47 (9)	21 (9)	21 (11)	9 (12)	9 (12)	8 (8)	
Other	93 (18)	14 (6)	24 (13)	21 (28)	9 (12)	39 (42)	
Total	515	222	185	75	73	94	
Legal							
Are you worried about the possibility that the results of a PG test may be passed to unauthorized persons?							
Worried	238 (44)	71 (43)	69 (44)	30 (46)	24 (37)	44 (51)	1.0
Not worried	190 (35)	65 (39)	60 (38)	24 (36)	21 (33)	20 (23)	
No opinion	112 (21)	30 (18)	29 (18)	12 (18)	19 (30)	22 (26)	
Total	540	166	158	66	64	86	
In case unfavorable test results should be disclosed, do you believe that you would be disadvantaged at work or job seeking?							
Yes	165 (31)	45 (27)	49 (31)	26 (39)	21 (35)	24 (28)	0.400
No	219 (41)	86 (51)	71 (45)	17 (26)	15 (25)	30 (35)	
Not sure	152 (28)	38 (22)	38 (24)	23 (35)	24 (40)	32 (37)	
Total	536	169	158	66	60	86	
Social							
In case of an unfavorable test result, do you believe you would feel “helpless” or “pessimistic”?							
Yes	174 (33)	63 (38)	40 (25)	26 (39)	18 (29)	27 (32)	0.170
No	265 (49)	69 (42)	93 (58)	35 (53)	32 (52)	36 (42)	
Not sure	98 (18)	34 (20)	25 (16)	5 (8)	12 (19)	22 (26)	
Total	537	166	158	66	62	85	
In case of an unfavorable test result, do you believe you would feel “different” or “inadequate”?							
Yes	152 (28)	51 (31)	32 (20)	25 (38)	19 (31)	25 (30)	0.100
No	268 (50)	84 (50)	95 (60)	32 (48)	27 (44)	30 (36)	
Not sure	115 (22)	31 (19)	31 (20)	9 (14)	15 (25)	29 (34)	
Total	535	166	158	66	61	84	

*ML&HS*, Molecular Life and Health Science

*Chi-square test, Bonferroni-adjusted *p* values

^a^Percentage (%)

**Table 9:** Students’ opinion regarding the confidentiality and data privacy in pharmacogenomic testing

	Model I	Model II	Model III
	OR (CI)	OR (CI)	OR (CI)
In case of an unfavorable test result, do you believe that you would feel “different” or “inadequate”?			
Q1			
Are you worried about the possibility that a pharmacogenomics test may reveal that you have additional risk factors for other diseases?	2.48 (1.34–4.60) *p* = 0.004	2.17 (1.15–4.09) *p* = 0.017	2.15 (1.13–4.10) *p* = 0.020

Model I: without adjustment; model II: adjusted to age and gender; model III: adjusted to age, gender, and level of education

Answer as a reference for Q1: yes

## Discussion

This is the first study that analyzed the level of awareness and attitude towards genetic tests, pharmacogenomics, and personalized medicine among students from several different universities in Bosnia and Herzegovina (BH). Our results showed that health and molecular life science students are generally aware of PG, and the level of awareness about personal genome testing companies appears to be similar between medicine and pharmacy students. However, students from the Faculty of Health Studies (FHS) seem to be less aware of these companies and less interested to employ PM as the novel healthcare model as compared to pharmacy, medicine, or genetics students. Although the respondents from the other non-health- and molecular life science-related studies are also generally aware of genes influence on their health, our results suggest that the level of their awareness about personal genome testing companies is significantly lower than that of their peers from medicine, pharmacy, and genetics.

Importantly, here we also demonstrated that about 40% of pharmacy students believe that PG should be an important part of their study curriculum and more than 60% of these students would like to continue their postgraduate education in the field of personalized medicine. This is in line with the recent study, which showed that the majority of students from the eight pharmacy schools in California were aware of pharmacogenomics, agreed that PG is important for the future pharmacist, and would be interested in a residency, fellowship, and/or career specializing PG [51]. However, Latif [8] reported that in the USA by 2005, PG was only being taught at a cursory level and highlighted the need to incorporate PG into the pharmacy curriculum. A recent survey of pharmacy students in California concluded that the presence of a stand-alone PG course did not impact student-perceived preparedness for a career in pharmacogenomics [51]. These findings are in accordance with the other studies including students from the medical schools in the UK [41] and USA [52], which also clearly indicated that inadequate education at undergraduate and postgraduate medical programs is an important obstacle to more broad use of PG. A recent study performed at the Stanford School of Medicine showed that almost all students taking a course in personalized medicine believed that physicians are not trained to interpret results of PG tests and thus are not able to effectively practice PM [52]. In line with this study, more than a third of the total number of students participating in our survey disagree that the curriculum of their study program is well designed to understand PG, suggesting that the majority of faculties do not have PG-related courses implemented in their curricula. Similar to our finding that 52% of pharmacy students disagree that their study program is well designed for understanding PG, half of the pharmacy students at the University of Minnesota also argued that their curriculum is not well designed to grasp pharmacogenomics [38]. Interestingly, in contrast to the pharmacy students from the University of Tuzla whose curriculum covers PG topics cursorily, usually not more than a week in a semester as a part of other coursework based on their current curriculum (http://frmf.untz.ba/web/bs/integrisani-i-i-ii-ciklus/), pharmacy students from the University of Sarajevo think that their curriculum is well designed to understand PG and PM. The Faculty of Pharmacy in Sarajevo implemented changes in their curriculum in 2012. Based on our knowledge and information available at the Faculty’s website (http://ffsa.unsa.ba/wp-content/uploads/2014/03/ECTS-katalog-2015.pdf), so far in BH only this Faculty included an elective course Pharmacogenomics and Personalized Therapy in the biomedical study course. In addition, pharmacy students from Sarajevo have a lot of subject education built into their other coursework. This is may be the reason why student pharmacists from Sarajevo had more positive attitude and future plans towards PG, while the majority of the pharmacy students from Tuzla disagree that PG should be an important part of their study curriculum.

Students of the Genetics and Bioengineering program at the International University of Sarajevo have special PG topics included in the syllabus of several undergraduate and graduate courses, including courses Pharmaceutical Biotechnology and Omics Technologies. Interestingly, more than 70% of GBE students agree that PG should be an important part of their study curriculum and more than half of them would like to continue their postgraduate education in the field of personalized medicine. Furthermore, as expected, in contrast to students from HS and MLS studies, half of students from other non-related study programs are not interested to continue their education in the field of pharmacogenomics and personalized medicine. Thus, in line with previous studies [38, 51], our results confirmed that if students do not gain enough PG knowledge during their studies, that would affect their attitudes towards PG as well as their future interest for this area of research or professional practice. Specifically, McCullough et al. [16] showed that pharmacists included in their study lacked the knowledge and self-confidence to act properly based on the results of PG testing. However, an education emphasizing medical applications of PG can significantly increase students’ knowledge and comfort in their PG practice. Recently, Pisanu et al. [53] investigated the discrepancy in PG education in Southeast Europe and recommended that PG should be thought as a stand-alone course or at least as a part of existing genetics courses. The lack of education and clinical guidelines appear to be among the major barriers perceived by participants towards the clinical application of PG [51].

It is expected that PG will continue to evolve over time and become one of the most relevant aspects of patient care. For this reason, it is of key importance to increase the number of professional practitioners in this new and expanding area of PG and to modify the current curricula to increase students’ knowledge and interests. The survey performed in the UK in 2008 found that typically 2–8 h of PG teaching were included in the pharmacology curricula of UK medical schools [41]. Previous studies have shown that incorporating active learning experiences in PG would increase student interests [54]. Additional innovative learning methods in PG have been recently adopted, such as Pharmacogenomics Education Program 3 (PharmGenEd™) open online courses [9] and personal genotyping [52, 55, 56]. When teaching PG through practical applications, students learn to use genetic information in the framework of medication management, allowing them to understand the significance of PG applications in clinical practice [51].

As our results indicate, BH students are interested to continue their education in PG. They would like to learn more about pharmacogenomics, its clinical examples and benefits, as well as about ELSI and future developments in this field. Students consider performing genetic tests in their future practice to optimize therapy for their patients as well as answering patients’ questions regarding PG and personalized medicine. Interestingly, our results suggest that the field of study significantly influences students’ wish to continue their education in this area. This is in line with the previous studies which indicated that healthcare students believe that PG is important for patient care [16, 40] and that they should have the knowledge to employ genetic tests results to optimize therapy and educate their patients [38].

Students participating in our survey have shown to be aware of different ethical aspects of genetic testing. Interestingly, our results demonstrated that the majority of students appear to be concerned about the patient’s privacy and data confidentiality, followed by other ethical issues, such as autonomy, trust, beneficence relating to incidental findings, racial issues, and stigma. The majority of participants in our survey believe that the physician, pharmacist, and genetic counselor should have an access to their PG information. If genetic information is inappropriately disclosed, individuals may suffer from embarrassment, stigma, and discrimination, and these issues are recently considered as the key aspects of respecting confidentiality [25]. This is increasingly a salient point with developments and prevalence of information and communication technology, especially in the context of health, with emerging EU regulations in context of data sharing. Information and tools that were previously accessible to physicians only under controlled clinical setting within the last decade have been made freely available through the increasing variety of the direct-to-consumer (DTC) genetic tests on the Internet and social networks, often without the public’s ability to understand the health risk information that are sold without genetic counseling [57, 58]. This issue is particularly important in the low- and middle-income countries, where the use of commercial genomics and DTC tests might not be adequately regulated yet.

Our results suggest that students from non-HS- and non-MLS-related faculties, including architecture, psychology, industrial, mechanical, electrical engineering, and others, are also generally aware of genes influence on their own health as well as about benefits of PM-based healthcare model. About half of these students agree that personalized medicine represents a new and promising healthcare model, and the majority of them would consider having a genetic test done to find out what illnesses they might develop in the future. However, their awareness of personal genome testing companies is lower as well as their readiness to contact a personal genome testing company and order a PG test, as compared to their peers from medicine, pharmacy, and genetics. Furthermore, these students from non-HS- and non-MLS-related faculties appear to be less aware regarding the potential ethical implications of PG testing, as compared to the students from medicine and pharmacy. These findings may indicate the significance of educating the public about genomics and its relevant bioethical implications. As recently suggested by Dressler et al. [59], roundtable discussions, a body of experts’ discussions, workshops, and symposia are needed to bring together key interdisciplinary stakeholders in academia, government, profit, and nonprofit organizations to create programs of genomic education for the public. Such efforts can lead to enhanced knowledge and widespread acceptance of PG.

Interestingly, our findings revealed that almost half of all respondents are worried about the possibility that PG test results may be passed to the unauthorized persons, and this opinion was shared similarly across different study programs. Students who are worried about the possibility that PG test may reveal that they have additional risk factors for other diseases would also feel “different” and “inadequate” in case of the unfavorable test results. Otherwise, about half of respondents would not feel “helpless” or “pessimistic,” nor they would not feel “different” or “inadequate.” This is in line with the previous study, which indicated that every individual would respond in a different way to the genetic test results, and it is considered essential for patients to have a proper counseling to help them understand the meaning and significance of the test results related to their own health [60, 61]. This also emphasizes the importance of sociological disciplines in public perceptions of pharmacogenomics and personalized medicine in order to examine and understand better the society’s needs, concerns, and attitudes towards the utilization of PG testing and its wider clinical implementation as well as to be an asset in instituting policies and regulating the use of genetic information. This is in line with the findings of the recent survey of the general public in Belgium on genetics and genetic testing [62], which indicated that recognizing the attitudes and concerns of the general public is the key in ensuring ethically reliable and socially acceptable application of new genetic technologies. Sociology students should be also approached to expand such studies of people’s reactions to genetics and PM/PG, where it would be pertinent to compare expected attitudes with actual attitudes upon receiving results of genetic tests [22].

An important strength of our study was that we recruited a variety of health science students across the nation within three different settings (medicine, pharmacy, health studies), genetics students, and students from other non-molecular life and non-health science programs. Another key aspect of our study was that, for the first time in BH, we have investigated students’ perceptions regarding their knowledge, skills, and attitudes towards pharmacogenomics and personalized medicine as well as their ethical, legal, and social implications. In addition, we compared opinions and attitudes of students who were exposed vs. students who were non-exposed to the PG course that further strengthens our results. Although our survey explored students’ interest in learning more about PG, we did not investigate which teaching tools students would favor in order to determine the most effective way to educate students in PG. Another limitation is that the survey which we designed assessed perceived (or self-reported) understanding and skills in pharmacogenomics and personalized medicine, with the limited possibility to evaluate actual students’ knowledge and capabilities. Lastly, survey tools that employ Likert scale are prone to central tendency bias due to selection of neutral answers. However, the potential impact of this type of bias on our results is probably small due to low percentage of neutral answers in the majority of our questions. Notwithstanding these limitations, our study will offer an important reference point for future comparative studies between different regions and countries as well as between different disciplines.

## Conclusions

Here we investigated for the first time students’ perceptions about pharmacogenomics and personalized medicine across the nation and various study programs that was, based on our knowledge, never studied before in Bosnia and Herzegovina. Our results show that most of the students participating in our survey, other than pharmacy students from Sarajevo, believe that they do not have well-designed curricula for understanding and practicing PG. The large number of students enrolled in molecular life and health sciences clearly expressed their wish to be more educated in this field. This implies the need for the development of study programs in the area of PG in order to equip future providers with the knowledge, skills, and attitude required to practice personalized medicine. This could also further highlight the need for increased genetic literacy education throughout high school levels throughout Europe and beyond. In order to accomplish this important goal, it would be pertinent to enhance collaboration between universities, healthcare institution, and governing bodies to incorporate more training and continued education topics related to pharmacogenomics and genetic testing. There could also be a potential here for increasing the number of interdisciplinary events or training between different disciplines, such as those highlighted in this study, both for students and for educators and researchers in order to discuss various forms of curriculum development. Thus, expanding the pharmacogenomic path of biomedical education represents an essential step for ensuring the widespread clinical implementation of personalized medicine.

## Additional files

Additional file 1: The survey questionnaire—the file describes the questionnaire that was employed in this study. (PDF 295 kb)

Additional file 2: Students’ awareness about pharmacogenomics—the table represents *p* values calculated with chi-square test between each faculty, based on the first question from Table 2. (PDF 50 kb)

Additional file 3: Levels of students’ awareness about genetic tests and pharmacogenomics—the table represents *p* values calculated with chi-square test between each faculty, based on the fourth question from Table 2. (PDF 204 kb)

Additional file 4: Students’ opinion regarding the study curriculum and their future plans in pharmacogenomics—the table represents *p* values calculated with chi-square test between each faculty, based on the first question from Table 4. (PDF 134 kb)

Additional file 5: Students’ opinion regarding the study curriculum and their future plans in pharmacogenomics—the table represents *p* values calculated with chi-square test between each faculty, based on the third question from Table 4. (PDF 134 kb)

Additional file 6: Students’ awareness and opinion regarding the ethical, legal, and social issues—the table represents *p* values calculated with chi-square test between each faculty, based on the first question from Table 8. (PDF 133 kb)

## Abbreviations

BH: Bosnia and Herzegovina
CI: Confidence intervals
DTC: Direct-to-consumer
ELSI: Ethical, legal, and social implications
FDA: Food and Drug Administration
FHS: Faculty of Health Studies
GBE: Genetics and Bioengineering
HS: Health Studies
IBM SPSS®23: IBM Statistical Package for Social Science, Version 23
MLS: Molecular and life sciences
OR: Odds ratio
PG: Pharmacogenomics
PharmGenEd™: Pharmacogenomics Education Program
PharmGKB: Pharmacogenomics Knowledgebase
PM: Personalized medicine
