# Supplementary material for: Perceptions of students in health and molecular life sciences regarding pharmacogenomics and personalized medicine
Source: Hum Genomics. 2018 Nov 14;12:50. doi: 10.1186/s40246-018-0182-2 (PMC6234656; doi:10.1186/s40246-018-0182-2)
Supplement: Supplementary file 1 — The survey questionnaire—the file describes the questionnaire that was employed in this study. (PDF 295 kb) [file 40246_2018_182_MOESM1_ESM.pdf]

Survey Description

Name of this research study is "*Genetic Testing and Personalized Medicine: Biomedical and Ethical Perspectives in Bosnia and Herzegovina*" and it is a part of the project initiated by the Genetics and Bioengineering Program at the Faculty of Engineering and Natural Science, International University of Sarajevo.

With a recent wide scientific breakthroughs and technological advancements in genetic testing, personalized medicine has the capacity to detect the onset of disease at its earliest stage, prevent the progression of disease, and optimize disease treatment.

In this survey we would like to investigate students' opinion about genetic testing and what are the obstacles to the popularization and clinical implementation of *"Personalized medicine"* in Bosnia and Herzegovina.

We appreciate your interest in this survey.

Instructions

This survey is anonymous and will be used for research purposes only. It will take approximately 15 minutes of your time. Please, answer all questions as honestly as you can. All your answers will be completely confidential. If for any reason, you do not feel comfortable answering any specific question, you can decline answering it.

Key definitions:

“Genetic testing" analyzes an individual’s genetic material that will help patients and their doctors to identify a person with predisposition for a particular disease; detect whether a person has a disease; identify the effectiveness and potential risk of side effects of a particular drug for an individual patient.

"Personalized medicine" refers to an innovative approach to the disease diagnosis and treatment that takes into account differences in people's genes, environment, and lifestyles.

“Pharmacogenomics“ is the study of association of individual’s genetic variation with drug response.

“Pharmacogenomic test” analyzes individual’s genetic material (DNA), in order to find out if they will benefit from a drug, require a different dose, or experience side effects. For example, the CYP450 test identifies variation in two genes, *CYP2D6* and *CYP2C19*, which are associated with metabolism of at least 50 commonly prescribed drugs. Variation in *CYP2D6* gene can result in low/slow/poor, normal/rapid/extensive, or ultra-rapid metabolism of some drugs. Those patients who metabolize drug slowly are at increased risk of having the drug remain in their blood for a prolonged period, which in turns increases their risk of adverse side effects. Ultra-rapid metabolizers may not achieve sufficiently high levels in their blood to have therapeutic effect since the drug is metabolized and eliminated too quickly.

By completing this survey I am giving my informed consent to participate in this research.

Survey Questionnaire

1. Mark your age

<19 19-26 26-40 41-50 51-60 >60

2. Mark your gender

Male Female

3. Mark your level of education

Less than highschool
BSc
PhD
Highschool
MSc
No answer

4. What is your family average monthly income?

<500 KM
1000-2000 KM
500-1000 KM
>2000 KM

5. What is your field of study?

Medicine
Genetics and Bioengineering
Health Studies
Other ____________________
Pharmacy

6. Have you been diagnosed with any of following diseases? You can choose multiple options.

Cardiovascular (heart problems, atherosclerosis, hypertension)
Metabolic diseases (diabetes, metabolic syndrome)
Oncology (any type of cancer)
Psychiatry (depression, anxiety)
Other ______________________
No

7. Did you ever take a drug that is used to treat any of the following diseases? You can choose multiple options.

Cardiovascular
Psychiatry
Metabolic disease (Diabetes)
Other ___________________
Oncology
I do not take drugs

8. Have you ever had an adverse drug reaction?

Yes
Don't know
No
I have never taken any medication

9. Have you ever found that a particular drug did not work for you?

Yes
Don't know
No
I have never taken any medication

10. To what extent do you think that genes influence your health?

Completely
Not at all
Moderately
Don't know

11. Would you consider having a genetic test done to find out what illnesses you might develop in the future?

Yes
No
Don't know

12. Have you heard about personal genome testing companies?

Yes
Don't know
No
Not sure

13. Would you consider contacting personal genome testing company and ordering a pharmacogenomic test for yourself?

Yes
Don't know
No
Not sure

14. If you know your genetic tendency to develop a disease, would you be ready to make necessary changes in your lifestyle, to reduce disease risk?

Yes
No
Maybe
Don't know

15. If a pharmacogenomic test revealed that prescribed drug would either be ineffective or cause severe side effects, would you take the drug anyway?

Take the drug anyway

Accept the test result, and not take the drug

Accept the test result, and take the drug only if the disease might be life-threatening

Not sure

16. Do you agree that personalized medicine represent a new and promising healthcare model?

Yes
No
Don't know

17. Pharmacogenomics should be an important part of my study curriculum.

Agree
Disagree
Neutral
No opinion

18. Do you think that curriculum of your study program is well designed for understanding pharmacogenomics?

Yes

Don't know

No

Not sure

19. In my future practice, I should be able to identify patients that could benefit from genetic testing.

Agree

Disagree

Neutral

No opinion

20. In my future practice, I should be able to answer patient's questions regarding pharmacogenomics and personalized medicine?

Agree

Disagree

Neutral

No opinion

21. In my future practice, I should be able to identify drugs that would require pharmacogenomics testing prior to their administration to the patient.

Agree

Disagree

Neutral

No opinion

22. Would you like to continue your postgraduate education (master, PhD, specialization) in the field of personalized medicine?

Yes

Don't know

No

Not sure

23. Which pharmacogenomics topics would you be interested to learn more about? You can choose multiple options.

Pharmacogenomics in general

Clinical examples of pharmacogenomics

Benefits of pharmacogenomics in clinical practices

Ethical, legal and social issues related to pharmacogenomics

Future development in pharmacogenomics

Other ___________________

24. How much money are you willing to spend to examine the effectiveness of a specific drug in your body using pharmacogenomic test?

<150 KM

150-500 KM

500-1000 KM

>1000 KM

Not sure

25. Are you aware of different ethical aspects of genetic testing?

Yes

No

Not sure

26. What ethical issues do you believe might be related to genetic or pharmacogenomic testing?

Patient privacy
Racial issues
Non-incidental findings
Data confidentiality
Stigma
Other

27. Are you worried about the possibility that the result of a pharmacogenomic test may be passed to unauthorized persons?

Very worried
Not worried
Slightly worried I don't know

28. Which of the following healthcare professionals should have access to your pharmacogenomic information? You can choose multiple options.

Physician
Genetic counselor
Psychologist
Pharmacist
Social worker
Other ________________
Nurse
Nutritionist

29. Are you worried about the possibility that a pharmacogenomic test may reveal that you have additional risk factors for other diseases?

Very worried
Not worried
Slightly worried
No opinion

---

30. In case an unfavorable test result should be disclosed, do you believe that you would be disadvantaged at work or job-seeking?

Yes
No
No opinion

31. In case of an unfavorable test result, do you believe that you would feel “helpless” or “pessimistic”?

Yes
No
No opinion

32. In case of an unfavorable test result, do you believe that you would feel “different” or “inadequate”?

Yes
No
No opinion

33. Do you believe that in the future pressure may be exerted on patients to agree to perform a pharmacogenomic test?

Yes
No
No opinion
