# Supplementary material for: Perceptions of students in health and molecular life sciences regarding pharmacogenomics and personalized medicine
Source: Hum Genomics. 2018 Nov 14;12:50. doi: 10.1186/s40246-018-0182-2 (PMC6234656; doi:10.1186/s40246-018-0182-2)
Supplement: Supplementary file 2 — Students’ awareness about pharmacogenomics—the table represents p values calculated with chi-square test between each faculty, based on the first question from Table 2. (PDF 50 kb) [file 40246_2018_182_MOESM2_ESM.pdf]

**Additional file 2: Table 2-q*1. Students' awareness about pharmacogenomics**				
Have you heard about personal genome testing companies?				
	Faculty of Medicine	Faculty of Health Studies	Genetics and Bioengineering	Non-ML&HS faculties
Faculty of Pharmacy	1.0**	0.025	0.770	<0.01
Faculty of Medicine		0.01	0.02	<0.01
Faculty of Health Studies			1.0	0.830
Genetics and Bioengineering				0.02

ML&HS-Molecular Life and Health Sciences; *q-question; **Chi square test, Bonferroni adjusted p values.
