# Supplementary material for: Perceptions of students in health and molecular life sciences regarding pharmacogenomics and personalized medicine
Source: Hum Genomics. 2018 Nov 14;12:50. doi: 10.1186/s40246-018-0182-2 (PMC6234656; doi:10.1186/s40246-018-0182-2)
Supplement: Supplementary file 3 — Levels of students’ awareness about genetic tests and pharmacogenomics—the table represents p values calculated with chi-square test between each faculty, based on the fourth question from Table 2. (PDF 204 kb) [file 40246_2018_182_MOESM3_ESM.pdf]

Additional file 3: Table2-q*4. Students' attitudes towards personalized medicine				
Do you agree that personalized medicine represent a new and promising health care model?				
	Faculty of Medicine	Faculty of Health Studies	Genetics and Bioengineering	Non-ML&HS faculties
Faculty of Pharmacy	0.06	<0.01	1.0	<0.01
Faculty of Medicine		1.0	0.330	0.310
Faculty of Health Studies			0.01	0.770
Genetics and Bioengineering				0.04

ML&HS-Molecular Life and Health Sciences; *q-question; **Chi square test, Bonferroni adjusted p values.
