# Supplementary material for: Perceptions of students in health and molecular life sciences regarding pharmacogenomics and personalized medicine
Source: Hum Genomics. 2018 Nov 14;12:50. doi: 10.1186/s40246-018-0182-2 (PMC6234656; doi:10.1186/s40246-018-0182-2)
Supplement: Supplementary file 4 — Students’ opinion regarding the study curriculum and their future plans in pharmacogenomics—the table represents p values calculated with chi-square test between each faculty, based on the first question from Table 4. (PDF 134 kb) [file 40246_2018_182_MOESM4_ESM.pdf]

Additional file 4: Table 4-q*1. Students' opinion regarding the study curriculum and their future plans in pharmacogenomics

Do you think that curriculum of your study program is well designed for understanding pharmacogenetics?

	Faculty of Medicine	Faculty of Health Studies	Genetics and Bioengineering	Non-ML&HS faculties
Faculty of Pharmacy	0.02	0.840	<0.01	<0.01
Faculty of Medicine		1.0	0.01	<0.01
Faculty of Health Studies			<0.01	<0.01
Genetics and Bioengineering				1.0

ML&HS-Molecular Life and Health Sciences; *q-question; **Chi square test, Bonferroni adjusted p values.
