# Supplementary material for: Perceptions of students in health and molecular life sciences regarding pharmacogenomics and personalized medicine
Source: Hum Genomics. 2018 Nov 14;12:50. doi: 10.1186/s40246-018-0182-2 (PMC6234656; doi:10.1186/s40246-018-0182-2)
Supplement: Supplementary file 6 — Students’ awareness and opinion regarding the ethical, legal, and social issues—the table represents p values calculated with chi-square test between each faculty, based on the first question from Table 8. (PDF 133 kb) [file 40246_2018_182_MOESM6_ESM.pdf]

Additional file 6: Table 8-q*1. Students' awareness and opinion regarding the ethical, legal, and social issues				
Are you aware of different ethical aspects of genetic testing?				
	Faculty of Medicine	Faculty of Health Studies	Genetics and Bioengineering	Non-ML&HS faculties
Faculty of Pharmacy	1.0	<0.01	0.610	<0.01
Faculty of Medicine		<0.01	1.0	0.02
Faculty of Health Studies			1.0	1.0
Genetics and Bioengineering				1.0

ML&HS-Molecular Life and Health Sciences; *q-question; **Chi square test, Bonferroni adjusted p values.
